# Alterations of bone proteins in medication‐related osteonecrosis of the jaw

**DOI:** 10.1111/eos.70003

**Published:** 2025-02-19

**Authors:** Andrea Schubert, Phillipp Brockmeyer, Philipp Kauffmann, Jan Wiegel, Florian Lautenbacher, Nicolai Miosge, Boris Schminke

**Affiliations:** ^1^ Department of Prosthodontics University Medical Center Goettingen Germany; ^2^ Department of Oral and Maxillofacial Surgery University Medical Center Goettingen Germany

**Keywords:** collagen 1, denosumab, MRONJ, RUNX2, zoledronate

## Abstract

Changes in the protein expression pattern of osteoblastic lineage cells from the alveolar bone (OLAB) during medication‐related osteonecrosis of the jaw (MRONJ) have rarely been investigated. This lack of information is partly because of the limited availability of healthy samples and the lack of human alveolar bone cell lines for research. The aim of the present study was to investigate the bone proteins collagen 1, runt‐related transcription factor 2 (RUNX2), and tumor necrosis factor ligand superfamily member 11 (RANKL). Furthermore, we established a cell lineage of OLAB suitable for the analyses of protein expression. We used immunohistochemistry to determine protein expression patterns in vivo. OLAB were treated during culture with zoledronate or denosumab and analyzed by immunocytochemistry and western blotting. Collagen 1 was decreased in vivo in patients with MRONJ and in vitro by denosumab. Zoledronate reduced the level of RUNX2 in vitro. However, RANKL was not significantly affected by zoledronate or denosumab. The results of the present study will help us elucidate the cellular mechanisms of MRONJ. Although culture of OLAB with zoledronate and denosumab significantly altered the protein expression patterns, future research is needed to examine the effects of bone scaffolds, biofilms, and additional cell types mimicking in vivo conditions.

## INTRODUCTION

The alveolar bone is a specialized tissue of the mandibula and maxilla, surrounding the teeth and forming the tooth sockets [1]. Healthy alveolar bone hosts osteocytes, osteoblasts, and osteoclasts that are embedded in a vascularized calcified extracellular matrix (ECM) that mostly consists of osteocalcin, osteopontin, and collagen types I, III, V, and VI [2, 3, 4]. Osteoblasts are responsible for maintaining the ECM [5], while osteoclasts degrade bone to initiate bone remodeling and mediate bone loss under pathological conditions through increased resorptive activity [6].

Patients suffering from osteoporosis [7], Paget's disease of the bone, or bone‐involving malignancies [8] often receive bisphosphonates or antiresorptive agents, such as denosumab, to stabilize bone remodeling and prevent the expansion of metastasis [9, 10, 11]. Bisphosphonates impair the ability of osteoclasts to form a ruffled border, to adhere to the bone surface, and to produce the protons necessary for continued bone resorption. They also reduce osteoclast activity by decreasing the development of osteoclast progenitor cells, and suppress recruitment of osteoclasts by promoting osteoclast apoptosis [12]. Denosumab is an anti‐tumor necrosis factor ligand superfamily member 11 (RANKL) IgG2 that binds to RANKL, inhibiting its interaction with tumor necrosis factor ligand superfamily member 11A (RANK). Tumor necrosis factor ligand superfamily member 11 usually converts progenitor cells into osteoclasts, thereby increasing bone resorption [13]. Use of bisphosphonates and other antiresorptive agents are widely accepted, but they can often lead to medication‐related osteonecrosis of the jaw (MRONJ) [14]. Medication‐related osteonecrosis of the jaw is characterized by a non‐healing jawbone exposed to the oral cavity or by bone that can be probed through an intraoral or extraoral fistula in the maxillofacial region, either of which has persisted for more than 8 weeks in patients with a history of, or ongoing use of, bisphosphonates, antiresorptive agents (such as denosumab), or an antiangiogenic agent. Patients with a prior history of radiation exposure to the head and neck region are excluded to be classified as MRONJ because bone necrosis in such patients may be caused by the radiation to which they were exposed [15]. The occurrence of MRONJ can be spontaneous after dentoalveolar surgical procedures or after trauma to the jaw. In 75% of patients with MRONJ, the mandible is affected. This is possibly because of the sparse blood supply and the compact bone structure of the mandible, especially when compared with the maxilla [16]. Medication‐related osteonecrosis of the jaw may be a refractory osteomyelitis rather than a true osteonecrosis, particularly when it develops after bisphosphonate use.

Medication‐related osteonecrosis of the jaw can be classified into stages according to clinical appearance. Stage 0 means that there is no clinical evidence of necrotic bone but rather nonspecific clinical findings, radiographic changes, and symptoms of the patient. Stage 1 is indicative of exposed and necrotic bone, or fistulae that connect to bone, in patients who are asymptomatic, without evidence of infection. Stage 2 involves exposed and necrotic bone, or fistulae that connect to bone, associated with infection, as evidenced by pain and erythema in the region of the exposed bone with or without purulent drainage. Patients with Stage 3 disease have exposed and necrotic bone or a fistula that connects to bone, and patients experience pain, infection, and exposed or necrotic bone extending beyond the region of the alveolar bone, resulting in one or more of the following conditions: a pathologic fracture, an extraoral fistula, oroantral/oronasal communication, or osteolysis extending to the inferior borderer of the mandible or sinus floor [17]. Depending on the stage, treatment for MRONJ may start conservatively with mouth rinses and antibiotics or can escalate to surgical debridement with tensionfree closure of the soft tissues. Ultimately, surgical therapy may include osteosynthesis or free microvascular tissue transfer [14].

The local pathological mechanisms in the alveolar bone during the development of MRONJ are still not completely understood. In this study, we investigated typical bone proteins, such as collagen 1 [18], runt‐related transcription factor 2 (RUNX2) [19], and RANKL [20], in vivo, and investigated the effects of zoledronate and denosumab, drugs frequently prescribed, on osteoblastic lineage cells from the alveolar bone (OLAB) in vitro.

## MATERIAL AND METHODS

### Tissue sources

Alveolar bone samples were obtained from five healthy patients whose premolars or third molars were extracted for orthodontic reasons (two male and three female patients; mean age 18.0 years). Samples of alveolar bone from eight patients with MRONJ were obtained during surgical debridement (five male and three female patients; mean age 71.8 years). All patients with MRONJ in this study were classified as having Stage 2 or 3 MRONJ. They underwent surgical treatment by the same surgeon and were followed up in our clinic every 8 weeks after suture removal. No recurrence of osteonecrosis occurred in these patients within the first 12 months after surgery. All patients provided written informed consent, consistent with the ethical regulations of our institution (file number: 22/1/05).

### Antibodies

Antibody immunoreactions without primary antibodies were performed as negative controls, and all experimental data are representative of three individual experiments. The antibodies used in this study are listed in Table 1.

**TABLE 1:** Antibodies used in the study.

Antigen	Clonality	Host	Supplier
RUNX2	Polyclonal	Rabbit	ab23981; Abcam
RANKL	Polyclonal	Rabbit	23408‐1‐AP; Proteintech
Neutrophil elastase	Polyclonal	Rabbit	ab21595; Abcam
Collagen 1	Polyclonal	Rabbit	R1038; OriGene
Denusomab	Polyclonal	Rabbit	E11‐1126B; EnoGene Biotech
Mouse IgG	Polyclonal	Goat	A9917; Sigma‒Aldrich
Rabbit IgG	Polyclonal	Goat	A0545; Sigma‒Aldrich
Alpha‐tubulin	Monoclonal	Mouse	T6199; Sigma‒Aldrich

RANKL, tumor necrosis factor ligand superfamily member 11; RUNX2, runt‐related transcription factor 2.

### Tissue preparation

For light microscopy, 15 mm × 15 mm samples of the alveolar bone were fixed in formalin at 4°C for 6 h, followed by washing for 15 min in running water. Briefly, decalcification was performed with 20% buffered ethylenediaminetetraacetic acid (EDTA) for 3 weeks. Dehydration and embedding in paraffin were performed using a Tissue Processor (165621‐46; Shandon Duplex), according to the manufacturer's instructions. Sections of 6 um thickness were cut using a Biocut Microtome (2035; Leica Instruments). The sections were transferred onto microscope slides (AAAA000001##12E; Thermo Scientific) and fixed by drying overnight at 37°C [21].

### Immunohistochemistry

The sections fixed onto microscope slides were subjected to 3 washes in xylene for 5 min each, followed by 2 washes in 100% ethanol for 10 min each, 2 washes in 95% ethanol for 10 min each, 2 washes in 70% ethanol for 10 min each, 2 washes in 50% ethanol for 10 min each, after which they were rinsed for 10 min in Tris‐buffered saline (TBS). Endogenous phosphatase activity was blocked by a 30‐min treatment at 20° with Universal Block (71‐00‐61; Seracare), followed by 3 washes of 10 min each in TBS. Epitope retrieval of the sections was achieved using ProTaqs (401603499; Quartett) for 20 min at 60°C, followed by 3 washes of 10 min each in TBS. The slides were treated for 5 min at 20° with 10 µg/mL of protease XXIV (P8038; Sigma‐Aldrich) using a volume of 0.5 mL. Blocking was performed with 1% bovine serum albumin (BSA) in TBS for 10 min at 20°, followed by 3 washes of 10 min each in TBS. Primary antibodies were applied at a dilution of 1:100 in TBS for 12 h at 20°C, followed by 3 washes of 10 min each in TBS. Visualization of antigens was performed using a HiDef Detection Alk Phos Polymer System (962D‐30; CellMarque), according to the manufacturer's instructions. The slides were digitally scanned (EasyScan One; Motic) at ×20 magnification with a resolution of 0.5 µm/pixel [21].

### Cell isolation and culture

All specimens were washed carefully with 5 mL Braunol (864219, Braun) 3 times for 1 min each and then 3 times with 5 mL PBS at 20°C. Afterwards, the healthy alveolar bone specimens were digested with 0.5 mg of dispase II (17105041, Thermo Fisher) and 1 mg of collagenase (17018029, Thermo Fisher) in 1 mL of Dulbecco's modified Eagle's medium (DMEM) for 12 h_at 20°C in a Falcon tube (10788561, Thermo Fischer). After digestion, the cells were released from their matrix using a 40 µm cell strainer (352340, Thermo Fisher) by allowing the cells to flow through the strainer and leaving the matrix fragments in the strainer. Then, 5 × 10^4^ cells were counted with a cellometer (Auto T4, Nexcelom Bioscience) according to manufactures instructions and transferred with a pipette to a 75 cm^2^ flask (83.1811.002, Sarstedt) containing 20 mL DMEM + GlutaMAX (21885‐025, Thermo Fisher) supplemented with fetal bovine serum (10270106, Thermo Fisher) to a final concentration of 10% and supplemented with gentamycin to a final concentration of 50 µg/mL. For the experiments, zoledronic acid (10355023, Accord) or denosumab (09199612, Amgen) was added once to the conventional cell culture medium to a concentration of 1 µM. The cells were treated with these antiresorptive agents for a duration of 5 days, after which they were harvested for further analysis [22].

### Immunocytochemistry

Cells at passage four were fixed with 2% paraformaldehyde in PBS for 15 min, followed by two washing steps with PBS for 10 min each wash. The cells were then permeabilized with 0.25% Triton X‐100 (X100‐5ML; Sigma‐Aldrich) in PBS for 10 min, followed by two additional washing steps with PBS for 10 min each wash. Blocking was performed with 1% BSA in PBS for 15 min at 20°C, after which the cells underwent two washing steps with PBS for 10 min each wash. Primary antibodies were diluted in 1% BSA in PBS and incubated for 1 h at 37°C according to the manufacturer's instructions, followed by two washing steps with PBS for 10 min each. Secondary antibodies conjugated to fluorochromes were applied at a dilution of 1:500, along with DAPI (71‐03‐01; Seracare) at a dilution of 1:1000 in PBS containing 1% BSA for 30 min at 37°C, followed by two final washing steps with PBS for 10 min each wash 20°C. The cells were then observed using fluorescence microscopy (BZ‐X700; Keyence) [21].

### Immunoblotting

A total of 1.5 × 10^5^ OLAB cells with and without the addition of antiresorptive drugs were dissolved in 30 µL of Laemmli sample buffer (300 mM Tris at pH 6.8, 22.5% glycerin, 9 % sodium dodecyl‐sulfate (SDS) and 0.03 % bromphenol blue) containing 10% ß‐mercaptoethanol and heated for 5 min at 95°C. Then, SDS‐PAGE at 20°C was performed with 6% acrylamide, in 1.25 mL buffer (15.1 g Tris in 250 mL H_2_O, pH 6.8), 0.055 mL SDS, 0.005 mL *tetramethylethylenediamine* (TEMED), 0.2 mL ammonium persulphate (APS) and 2.7 mL H_2_O in the stacking gel by applying 10 mA and 8% acrylamide in 1.4 mL buffer (45.4 g Tris in 250 mL H_2_O, pH 8.9), 0.055 mL SDS, 0.005 TEMED, 0.25 mL APS and 2 mL H_2_O in the separation gel by applying mA. The running buffer (30.3 g Tris, 142.5 g Glycin, 50 mL SDS, filed up to 1 L with H_2_O) is diluted 1:5 with H_2_O. After SDS‐PAGE, the separated proteins were blotted onto an Immobilon‐P Transfer Membrane (PVH07850; Merck Millipore) at 350 mA for 1 h in a transfer tank with cooling by running tab water. General detection of the proteins was performed by Coomassie blue (LC6065, Thermo Fischer) was performed according to the instructions of the manufacturer. After destaining, the membranes were blocked with Tris‐buffered saline containing 0.1% polysorbate 20 (TBS‐T) and 5% milk powder for 1 h, followed by five washing steps, 10 min each, in TBS‐T at 20°C. Then, the primary antibodies were dissolved in TBS‐T containing 5% milk powder according to the manufacturer's instructions and incubated for 12 h at 4°C. Again, five washing steps, 10 min each, in TBS‐T at 20°C were performed. The membranes were then incubated with secondary antibodies for 2 h at 20°C, followed by five washes, 10 min each, in TBS‐T at 20°C. Visualization of the proteins was achieved by applying WesternBright Sirius HRP substrate (K‐12043‐D10; Advansta) [21].

### Statistical analysis

We report representative data from at least three independent experiments performed on three biological replicates of healthy alveolar bone samples and on eight samples from patients with MRONJ. Tissue slides were digitized using a slide scanner (EasyScan One, Motic) at 20 x magnification and 0.5 um/pixel resolution. For semiautomated, semiquantitative immunohistochemical analysis, we used the image analysis software QUPATH (open‐source software; The Queen's University of Belfast). In each case, three different regions of interest (ROls) were digitally defined. The ROls in patients with MRONJ were selectively placed in areas on the slides that still exhibited residual tissue architecture, without complete necrosis. In this way, nonspecific false‐positive immunoreactions were not included in the evaluation. Each ROI was approximately 1 cm^2^ in size. The QUPATH positive detection algorithm was applied within each ROl. Using the default settings of the software, the immunohistochemically labeled marker proteins (neutrophil elastase, collagen 1, RUNX2, and RANKL) were semiautomatically scored based on the amount of positive staining per mm^2^ of bone. The mean values of the three different ROls were calculated for statistical analysis. These values were tested for a normal distribution using the Shapiro‐Wilk test. As all the data were normally distributed, group comparisons were performed using two‐tailed Student's t‐tests. All statistical analyses were performed at a significance level of α = 5% using PRISM 9.5 (GraphPad). A value of p ≤ 0.05 was considered to indicate statistical significance [23].

Exposed x‐ray films were scanned and then digitized. Densitometry was performed for quantification of protein in immunoblot lanes using IMAGEJ (open‐source software, National Institutes of Health). Alpha‐tubulin was used for normalization of protein loading. The immunoblotting results are reported as mean and standard deviation; numbers indicate fold changes. After testing for a normal distribution, we performed Student's t‐tests. Pearson correlation coefficients were calculated. A value of p ≤ 0.05 was considered to indicate statistical significance [21].

## RESULTS

For the first time, to the best of our knowledge, in vivo studies were performed comparing expression of alveolar bone proteins in healthy patients with those in patients with MRONJ. The results were then further verified in vitro by exposing OLAB to the antiresorptive drugs zoledronate and denosumab.

### Histochemistry of healthy alveolar bone and MRONJ samples in vitro

**FIGURE 1:**
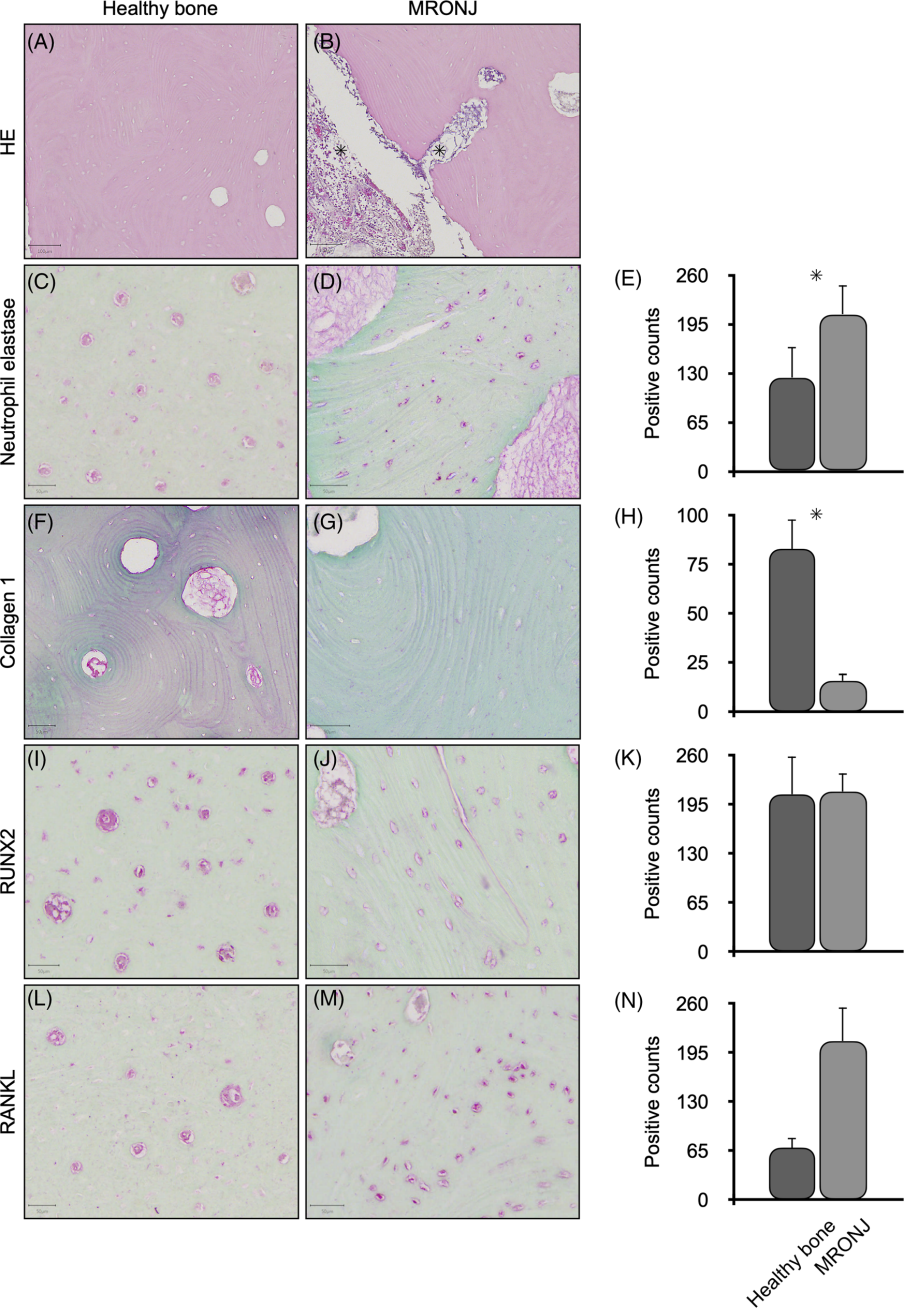
Histochemistry and immunohistochemistry of alveolar bone from healthy patients (left panel) and from patients with medication‐related osteonecrosis of the jaw (MRONJ) (middle panel). Statistical analysis is given in the right panel. Hematoxylin‐eosin (HE) staining of healthy alveolar bone (A) and of alveolar bone from patients with MRONJ (B): necrotic bone areas are indicated (with ★). Neutrophil elastase staining of healthy alveolar bone (C) and of alveolar bone from patients with MRONJ (D). Statistical analysis (E) demonstrated significantly more neutrophil elastase in alveolar bone from patients with MRONJ (marked with ★). Staining of collagen 1 in healthy alveolar bone (F) and in alveolar bone from patients with MRONJ (G). Statistical analysis (H) revealed significantly more collagen 1 in healthy alveolar bone (marked with ★). Staining of runt‐related transcription factor 2 (RUNX2) in healthy alveolar bone (I) and in alveolar bone from patients with MRONJ (J). There was no statistically significant difference in expression of RUNX2 between alveolar bone from healthy patients and alveolar bone from patients with MRONJ (K). Staining of RANKL in healthy alveolar bone (L) and in alveolar bone from patients with MRONJ (M). There was no statistically significant difference in expression of RANKL between alveolar bone from healthy patients and alveolar bone from patients with MRONJ (N). The scale bar equals 50 µm.

Using hematoxylin‐eosin histochemistry, we were able to identify the areas of necrosis in samples from patients with MRONJ (Figure 1B); by contrast, no necrosis was found in healthy samples of alveolar bone (Figure 1A). Neutrophil elastase was detected in cells as well as in the ECM by immunohistochemistry, and was present at significantly higher levels in alveolar bone samples from patients with MRONJ (Figure 1E), especially in areas of necrosis (Figure 1D), than in healthy alveolar bone samples (Figure 1C). The opposite was seen for the quantities of collagen 1 in the ECM (Figure 1H). The healthy alveolar bone samples exhibited significantly more collagen 1 (Figure 1F) than the alveolar bone samples from patients with MRONJ (Figure 1G). Although RUNX2 (Figure 1 I—K) and RANKL (Figure 1L—N) were detected in cells of both healthy alveolar bone and alveolar bone from patients with MRONJ, no significant differences were detected. No histopathological differences were found between alveolar bone samples from patients with MRONJ clinical stage 2 and those from patients with MRONJ clinical stage 3.

### Immunocytochemistry results

**FIGURE 2:**
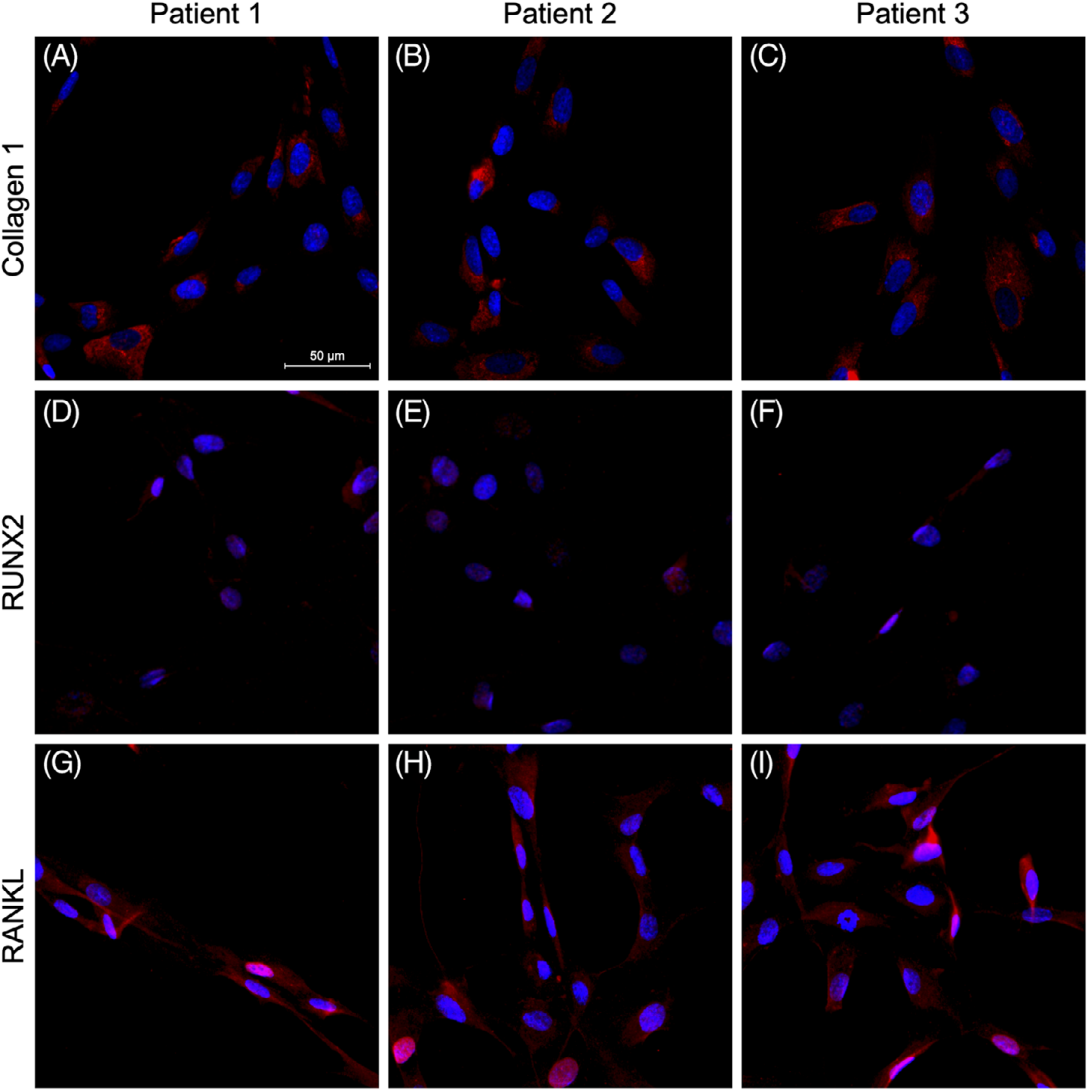
Immunocytochemistry for collagen 1, runt‐related transcription factor 2 (RUNX2), and tumor necrosis factor ligand superfamily member 11 (RANKL) in osteoblastic lineage cells from the alveolar bone (OLAB) from three patients with healthy alveolar bone. The OLAB cells from all three patients expressed collagen 1 in the cytoplasm (A—C). RUNX2 was present in the cytoplasm and nucleus in all patients (D—F). RANKL was present in the cytoplasm and nucleus in all patients (G—I). The scale bar equals 50 µm.

To transfer the results from the tissue analyses to the cell culture, OLAB cells were isolated from three healthy patients. However, it is not possible to obtain vital cells from samples of patients with MRONJ. In all three healthy patients (Figure 2), collagen 1 was detected in the cytoplasm (Figure 2A–C), and small amounts of RUNX2 (Figure 2D–F) and of RANKL (Figure 2G–I) were detected in the cytoplasm and nucleus, as previously shown in Figure 1 from in vivo experiments.

### Influence of zoledronate and denosumab on OLAB cells

**FIGURE 3:**
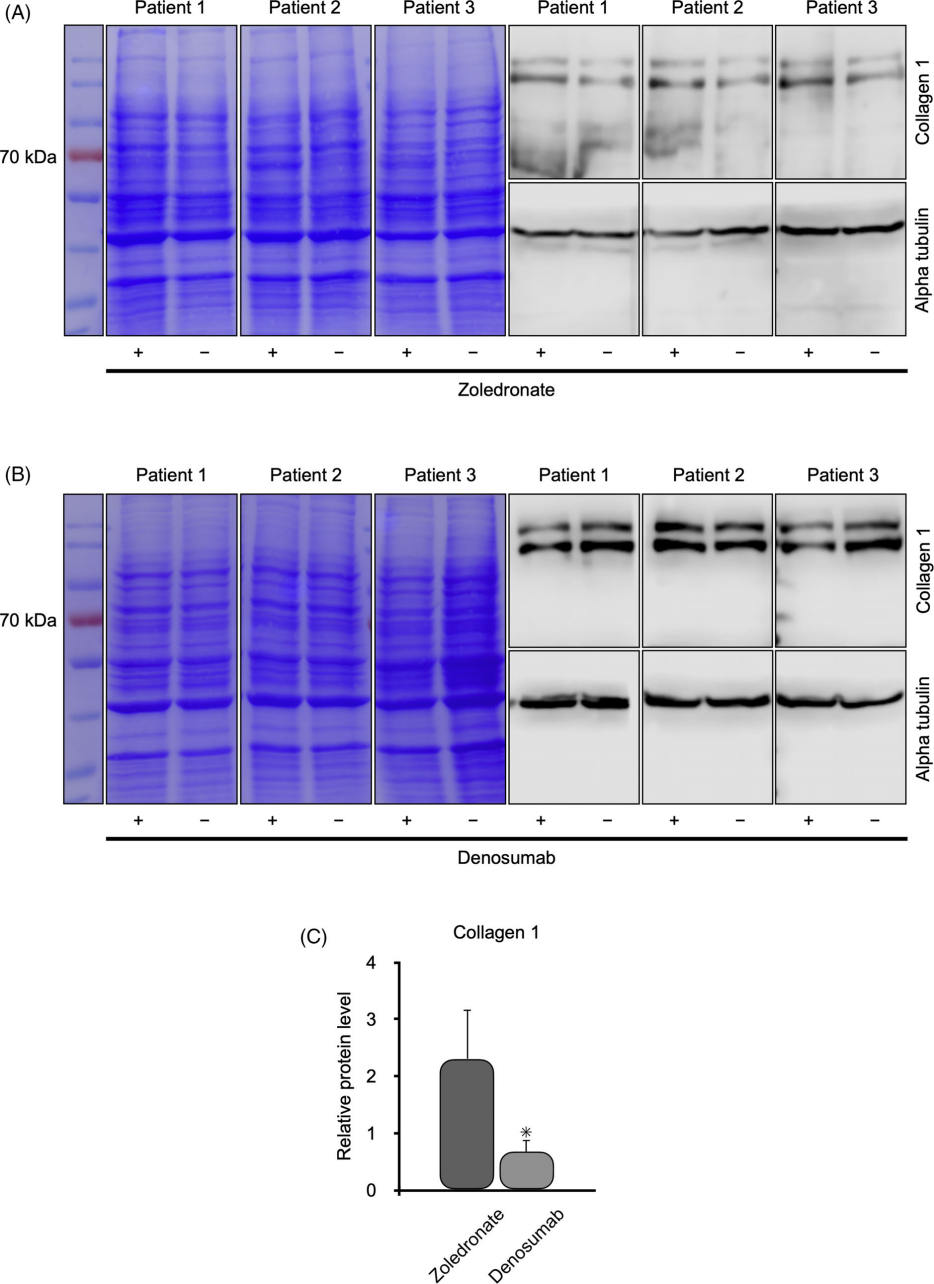
Western blot analysis for collagen 1 in osteoblastic lineage cells from the alveolar bone (OLAB) obtained from three patients with healthy alveolar bone. The OLAB cells were treated with (+) or without (−) zoledronate (A) or denosumab (B). The protein ladder is on the left. Coomassie brilliant blue staining showed proper separation of the proteins, and the expression of α‐tubulin confirmed that an equal concentraion of protein was loaded into each well of the gels. Statistical analysis showed significantly lower levels of collagen 1 in OLAB cells treated with denosumab (C).

**FIGURE 4:**
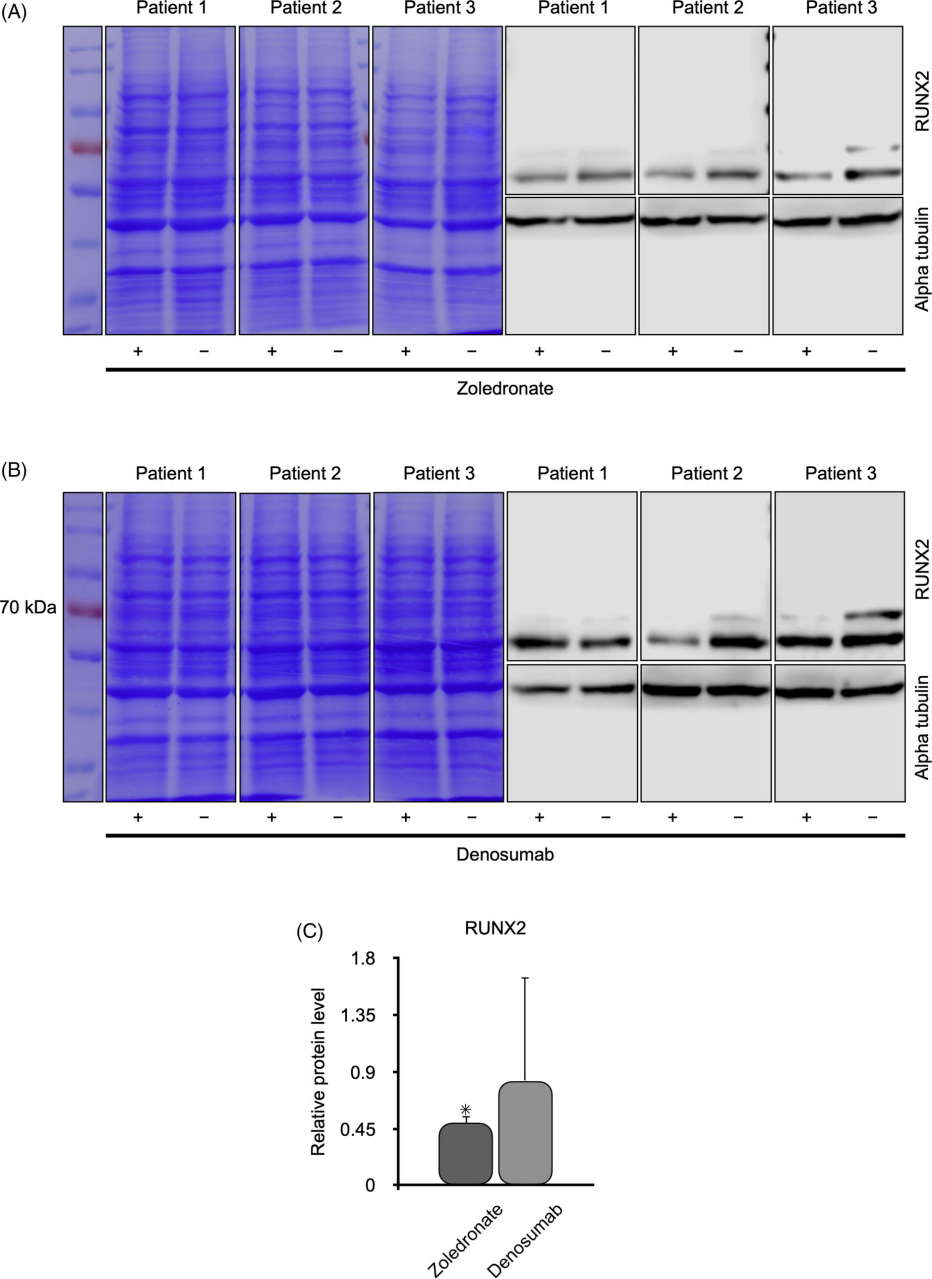
Western blot analysis of runt‐related transcription factor 2 (RUNX2) in osteoblastic lineage cells from the alveolar bone (OLAB) obtained from three patients with healthy alveolar bone. The OLAB cwere treated with (+) or without (−) zoledronate (A) or denosumab (B). The protein ladder is on the left. Coomassie brilliant blue staining showed proper separation of the proteins, and the expression of α‐tubulin confirmed that an equal concentration of protein was loaded into each well of the gels. Statistical analysis showed significantly lower levels of RUNX2 in OLAB cells treated with zoledronate (C).

**FIGURE 5:**
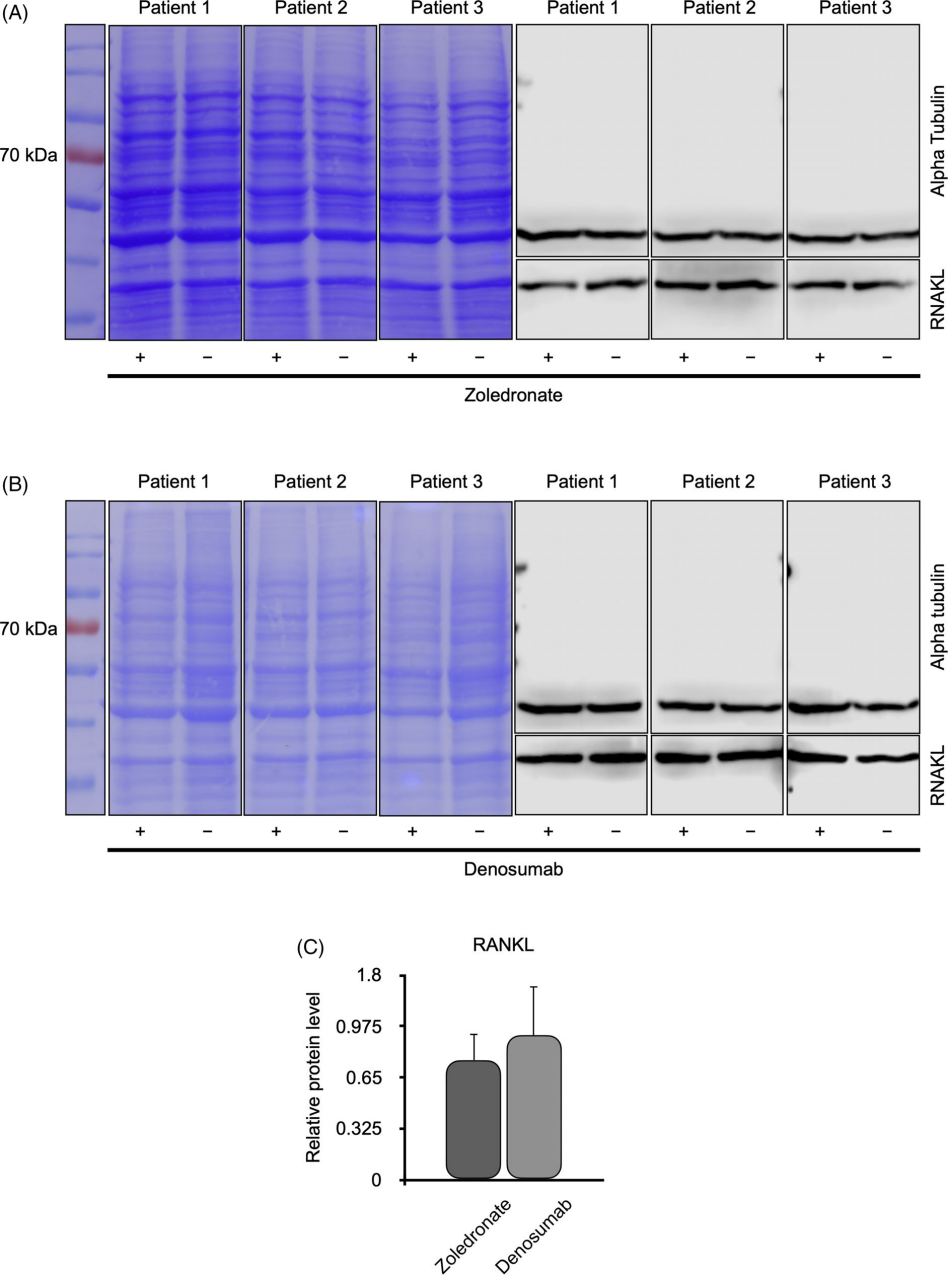
Western blot analysis of tumor necrosis factor ligand superfamily member 11 (RANKL) in osteoblastic lineage cells from the alveolar bone (OLAB) obtained from three patients with healthy alveolar bone. The OLAB cells were treated with (+) or without (−) zoledronate (A) or denosumab (B). The protein ladder is on the left. Coomassie brilliant blue staining showed proper separation of the proteins, and the expression of α‐tubulin confirmed that an equal concentration of protein was loaded into each well of the gels. Statistical analysis showed no significant effects of either zoledronate or denosumab on expression of RANKL by OLAB (C).

Immunoblotting was used to demonstrate the effects of the antiresorptive agents zoledronate and denosumab on OLAB cells from three healthy patients on the synthesis of collagen 1, RUNX2, and RANKL (Figures 3, 4, and 5).

Zoledronate and denosumab had different effects on the synthesis of collagen 1. We found that OLAB produced more collagen 1 in the presence of zoledronate (Figure 3A) than in its absence. The opposite effect was observed for denosumab (Figure 3B), namely, the amount of collagen 1 was reduced when denosumab was present. However, the increase of collagen 1 in the presence of zoledronate was not statistical significant and was only observed for samples from patients treated with denosumab (Figure 3C).

The synthesis of RUNX2 was significantly decreased in all samples of OLAB treated with zoledronate (Figure 4A), whereas denosumab tended to increase the level of RUNX2 (Figure 4B). The effect of denosumab was not statistically significant (Figure 4C).

Zoledronate (Figure 5A) and denosumab (Figure 5B) influenced the levels of RANKL in OLAB without statistical significance. There was a slight tendency toward a reduction in RANKL in OLAB in the presence of zoledronate and denosumab, but the difference was not statistically significant (Figure 5C).

## DISCUSSION

In the present study, we investigated the protein expression patterns of OLAB cells in vivo and in vitro. The samples from patients with MRONJ showed high concentrations of neutrophil elastase, a typical sign of inflammation [24] of the exposed bone in the oral cavity, which plays a part in degrading the ECM of bone tissue and enhancing osteoclast formation [25]. Collagen 1, the main collagen in bone tissue [2], was decreased in all samples from patients with MRONJ, indicating the loss of ECM, as known for peri‐implantitis [26], periodontitis [22], and osteoporosis [27]. By contrast, staining of collagen 1 in healthy alveolar bone samples was particularly strong, as expected based on previous data [5]. The changes in collagen 1 content observed could potentially be clinically validated through measurements of C‐terminal telopeptide crosslinks of type 1 collagen in serum tests, which may serve as a parameter for the progression of MRONJ or predict patient responses to antiresorptive therapies [28, 29]. While significantly reduced RUNX2 levels were previously observed in a mouse model [30], the staining of RUNX2 in cells embedded in the ECM showed a typical pattern for bone tissue [31] and demonstrated no difference to osteocytes in the ECM of healthy bone tissue compared with MRONJ. Although RANKL is one of the main regulators of the degradation of necrotic bone by osteoclasts [32], no significant differences in the levels of RANKL were found between osteocytes from healthy bone tissue and those from MRONJ, in vivo. The in vitro results were confirmed at the in vivo level by immunocytochemistry staining for collagen 1, RUNX2, and RANKL in OLAB cells, as previously described for a pool of bone cells [33].

For the first time, we describe cell‐specific protein expression patterns and specific changes in these patterns in OLAB cells upon treatment with zoledronate and denosumab. Surprisingly, zoledronate and denosumab had contrasting effects on the levels of collagen 1 protein in OLAB cells. Zoledronate seemed to upregulate collagen 1. Osteoprotegerin is known to upregulate the expression of ECM bone tissue components, such as collagen 1, by exposing gingival fibroblasts to bisphosphonates in vitro [34]. However, denosumab reduced the amount of collagen 1 in OLAB cells, and a low level of collagen 1 is known to be involved in degenerative bone diseases [27]. The level of RUNX2, a major regulator of osteoblast differentiation and transcription factor of bone ECM [35], was reduced by zoledronate and denosumab. Zoledronate also decreased the level of RUNX2 in MC3T3‐E1 cells, a model for the osteoblastic phenotype [36, 37]. The modulation of RUNX2 activity through binding adapter proteins, such as Ras‐related protein Rab‐5C (RAB5C), could represent a promising therapeutic approach to compensate for the in vivo loss of collagen type I as a structural protein in the jawbone of patients with MRONJ [38]. As RUNX2 is a growth factor, systemic therapy should be avoided, and local application options should be considered, especially given the role of RUNX2 in neoplasms, particularly in oral squamous cell carcinoma [39].

Interestingly, denosumab promotes osteogenic differentiation in mesenchymal stem cells by upregulating the expression of collagen 1, RUNX2, and alkaline phosphatase [40], findings in contrast to those for OLAB cells in the present study. Denosumab, a fully human monoclonal antibody directed against RANKL [41], and zoledronate reduced the level of RANKL in OLAB cells. The effect on bone like cells of a zoledronate‐induced reduction of RANKL is already known [42], while a direct reduction in RANKL levels due to the effect of denosumab is currently undetected and needs further investigation.

Alterations in collagen 1, RUNX2, and RANKL were investigated in vivo and in vitro in healthy patients as well as in patients with MRONJ. We stimulated MRONJ‐like conditions in vitro by the supplementation of OLAB cells with zoledronate or denosumab, whereas, in vivo, MRONJ is triggered by multiple factors, such as exposure of bone tissue to the oral cavity, the local microbiome, and the lack of an immune response in bone tissue. Therefore, our experimental procedures are not accurate simulations of in vivo MRONJ. Nevertheless, exposure to zoledronate and denosumab drastically altered the protein expression patterns of OLAB cells, even in this experimental approach. Further investigations, including bone scaffolds [43], mechanical forces [44], complex biofilms, and immune interactions [45], will be required to translate our findings to in vivo conditions. Recruitment of other cell types, such as oral fibroblasts, may be necessary to promote the regenerative effects of MRONJ [46]. Only then can transfer to a suitable animal model be considered, as is the case for tooth extraction‐related MRONJ in rodents [47].

The data presented here provide the first indication that collagen 1 and RUNX2 play crucial roles in the pathogenesis of MRONJ. The present study will help improve our understanding of the pathological mechanisms of MRONJ and may aid in the elucidation of new treatment options that focus on influencing cellular mechanisms. Remarkably, we established a pool of human oral cell lines from alveolar bone that will be available for future in vitro research.

## AUTHOR CONTRIBUTIONS

**Conceptualization**: Boris Schminke and Andrea Schubert; **Methodology**: Jan Wiegel, Florian Lautenbacher, Boris Schminke, Andrea Schubert, Phillipp Brockmeyer and Philipp Kauffmann; **Software**: Jan Wiegel and Phillipp Brockmeyer; **Validation**: Phillipp Brockmeyer, Andrea Schubert, Boris Schminke, Philipp Kauffmann and Nicolai Miosge; **Formal analysis**: Philipp Kauffmann, Boris Schminke, Phillipp Brockmeyer and Andrea Schubert; **Investigation**: Jan Wiegel, Florian Lautenbacher, Boris Schminke and Andrea Schubert; **Resources**: Nicolai Miosge; **Data curation**: Jan Wiegel, Florian Lautenbacher, Boris Schminke, Andrea Schubert and Phillipp Brockmeyer; **Writing—original draft preparation**: Andrea Schubert and Boris Schminke; **Writing—review and editing**: Andrea Schubert and Boris Schminke **Visualization**: Boris Schminke and Phillipp Brockmeyer; **Supervision**: Nicolai Miosge and Philipp Kauffmann; **Project administration**: Nicolai Miosge; All authors have read and agreed to the published version of the manuscript.

## CONFLICT OF INTEREST STATEMENT

The authors have no conflicts of interest related to this article.
